# Split potassium application delays senescence and increases grain yield in winter wheat grown on sandy and silt loam soils

**DOI:** 10.3389/fpls.2025.1599296

**Published:** 2025-05-22

**Authors:** Yongbing Wang, Xiaoxiao Yin, Xin Wang, Muhammad Fraz Ali, Xiang Lin, Shubo Gu, Yong Han, Dong Wang

**Affiliations:** ^1^ State Key Laboratory for Crop Stress Resistance and High-Efficiency Production, College of Agronomy, Northwest A & F University, Xianyang, Shaanxi, China; ^2^ College of Agronomy, Shandong Agricultural University, Taian, Shandong, China; ^3^ Wheat Research Institute, Anyang Academy of Agricultural Sciences, Anyang, China

**Keywords:** winter wheat, split K application, senescence, grain weight, yield

## Abstract

**Background:**

The judicious application of potassium (K) fertilizer plays a critical role in increasing potassium use efficiency, leaf photosynthesis capacity, and winter wheat yield. However, there is no unified conclusion on the yield-increasing effect of split K fertilizer application. In addition, the response mechanism of winter wheat to split K application across different soil types remains unclear.

**Aims:**

The aim of this study was to investigate the mechanism underlying the effects of split K application on winter wheat yield across different soil types and to provide a basis for optimized and judicious K fertilization in the Huang-Huai-Hai plain (3HP).

**Methods:**

A two-year field experiment with winter wheat from 2016 to 2018 on silty and sandy loam, using three K application levels (K0, no K; K1, 96 kg ha^-1^; and K2, 120 kg ha^-1^) and two methods (T1, 100% basal application, and T2, 50% basal application + 50% topdressing at jointing).

**Results:**

The split K application increased the K and nitrogen (N) accumulation of winter wheat plants compared to a single application. It also enhanced the flag leaf SPAD value, net photosynthetic rate (Pn), superoxide dismutase (SOD) activity, catalase (CAT) activity, and soluble protein content after flowering, while decreasing the malondialdehyde (MDA) content in both soil types. Additionally, split K application improved the grain-filling rate at 25 days after flowering, prolonged the active grain-filling period (*D*) and the actual filling period (*T3*), and enhanced the 1000-grain weight, grain yield, agronomic efficiency, and partial factor productivity of K fertilizer. Moreover, in sandy loam soil, the split K application was more effective in improving the SPAD value, Pn, Plant N and K accumulation, 1000-grain weight, yield, and agronomic efficiency of K fertilizer compared to silty loam soil.

**Conclusion:**

This study provides a basis for region-specific and soil-tailored potassium fertilizer management strategies, thereby optimizing resource utilization.

## 1 Introduction

Winter wheat is one of the most important food crops in many countries, including China (Qin et al., 2015), and it is a primary component of the diet, providing essential calories and protein (Li et al., 2019). With the rapid growth of the global population, ensuring a steady increase in the production of major food crops, such as wheat is critical to maintaining global food security. The Huang Huai Hai plain (3HP) is the central winter wheat-producing region in China and accounts for 70% of the total wheat production (Xiao et al., 2020). In this region, the main focus is placed on nitrogen (N) as the primary fertilizer for wheat production, while phosphorus (P) is considered as secondary fertilizer (Huang et al., 2017). However, potassium (K) fertilizer receives relatively low attention in wheat cultivation, as it has traditionally been assumed that the soil contains sufficient K for wheat production (Hou et al., 2023). Therefore, the main focus of research is that the N fertilizers play a significant role in improving crop growth and yield, and there has been a lack of systematic research on other elements that play an irreplaceable role in wheat growth and development.

K is an essential macronutrient involved in a wide range of biochemical, phenological, and physiological processes in plants (Hawkesford et al., 2012; Pandey and Mahiwal, 2020), making it indispensable for the growth and development of winter wheat (Johnson et al., 2022). The availability of K in soil is constrained by fixation and leaching, with the latter posing a significant threat to soil fertility, particularly in sandy soils under high rainfall conditions, which exacerbates K deficiency and reduces crop yield (Römheld and Kirkby, 2010; Das et al., 2022). The distribution of K in soil is governed by various physical and chemical properties, including cation exchange capacity, soil pH, particle size and quantity, and the presence of K-bearing minerals. K deficiency significantly reduces stomatal conductance in leaves (Jin et al., 2011; Yang et al., 2022), limiting carbon dioxide influx into leaves (Imtiaz et al., 2023) and decreasing the activity of the key photosynthetic enzyme *Rubisco* (Hu et al., 2023), thereby reducing the net photosynthetic rate. Optimal K application can activate enzymes (Hasanuzzaman et al., 2018), promote protein synthesis (Zörb et al., 2014; Johnson et al., 2022), enhance photosynthesis (Rawat et al., 2022), improve crop quality, and enhance stress tolerance (Wang et al., 2013; Oosterhuis et al., 2014), ultimately leading to increased crop yield (Zhan et al., 2016; Sedri et al., 2021; Zhao et al., 2022), which is critical for the growth of winter wheat.

In North China Plain, when the soil available K content is 150–180 mg kg^-1^, reasonable fertilization can help wheat to achieve relatively high yields (Ma et al., 2022). In sandy loam fluvo-aquic soil with an available K content of 66.93 mg kg^-1^, applying 120 kg ha^-1^ of K fertilizer significantly increased the number of grains per spike and thousand-grain weight of wheat, leading to higher yields. Similarly, in sandy loam soil (available K: 94.03-97.06 mg kg^-1^) and loamy sand soil (available K: 116.48-118.91 mg kg^-1^), split application of 120 kg ha^-1^ K fertilizer exhibited the optimal yield-increasing effect (Hu et al., 2021). These findings suggests that optimizing K fertilizer rates and management practices based on soil K supply and texture can achieve high-yield and efficient wheat cultivation.

In Chinese wheat-producing regions, K fertilization has been limited due to the high soil K levels reported in the 1980s (Zhou et al., 2007). However, continuous wheat-maize cropping with unbalanced fertilization has rapidly depleted soil K (Wang et al., 2024), leading to significant winter wheat yield losses. Although the use of K fertilizer is now being adopted, it is typically applied in a single dose. In contrast, split applications can reduce leaching losses, improve K use efficiency (KUE) (Römheld and Kirkby, 2010), and promote K accumulation in wheat plants (Sharma et al., 2022). Compared to single application, split K application offers several advantages. For example, Lu et al. (2014) reported that split application of K resulted in higher kernel weight, grain yield, and grain quality, all of which were improved synchronously. In rice, a significant increase in grain yield was observed due to the combined use of N and K, resulting from an increase in spikelets per panicle, and this approach not only boosted yield but also enhanced overall crop performance. In another study, Zhang et al. (2023) reported that under the same N application level, split K application significantly enhances N use efficiency (NUE) in winter wheat, with grain yield remaining substantially higher even with a 40% reduction in N (Zhang et al., 2023). The scientific community remains divided on the effect of K fertilizer application strategies in wheat. Some studies suggest that a single basal dose of K fertilizer minimally influence spike number, whereas split applications can boost yield by improving grain weight (Lu et al., 2014). However, other studies argue that split K application enhances grain yield by increasing both grain number per spike and 1000-grain weight (Hu et al., 2021; Sharma et al., 2022). Given the prevalence of severe K deficiency in sandy and silty loam soils (Andersson et al., 2007; Pettigrew, 2008; Grzebisz et al., 2013), research on the yield benefits of split K application in these two soil types remains limited, and the mechanisms underlying these yield improvements are still not well understood.

To study the yield-increasing mechanism of split K application, there is need to pay attention to the leaf senescence process. In the absence of exogenous stress input, leaf senescence mainly depends on leaf age and plant developmental stage (Zentgraf et al., 2004). The flag leaf is one of the crucial photosynthetic organs in wheat, playing a significant role in wheat grain yield. Nearly 50% of the assimilates produced during grain filling originate from the photosynthetic products of the flag leaves (Deng et al., 2018). However, the senescence of flag leaves can be triggered and regulated by various abiotic stresses. The supply of exogenous K can alleviate oxidative stress, increase antioxidant enzyme activity and the production of ROS scavengers, and interact with other molecules to promote crop growth and delay crop senescence (Du et al., 2019; Johnson et al., 2022). Some studies have shown that K deficiency can induce leaf senescence (Li et al., 2012; Wang et al., 2012; Du et al., 2019), while K surplus can also affect leaf senescence (Santos, 2001). However, it is still unclear whether the effects of the split K application on the senescence of winter wheat flag leaves are consistent in different soils.

In the 3HP, soil K status varies widely (He et al., 2015), with current production systems heavily reliant on one-time basal K application. This practice restricts effective K supply during critical K-demanding growth stages, while overlooking the interactive effects between soil types and K fertilizer management strategies on nutrient absorption and yield formation. As previously mentioned, split K application has been demonstrated as crucial for improving winter wheat yields and fertilizer use efficiency (Hu et al., 2021; Zhang et al., 2023). However, the mechanistic understanding of yield enhancement from split applications across different soil types remains insufficient, particularly regarding soil-specific nitrogen and potassium uptake dynamics and leaf senescence responses to split applications. In this context, optimizing K fertilizer management for different soil types is crucial. In this study, field experiments were conducted to study the interactive effects of split K in high fertility silty loam and low fertility sandy loam on winter wheat grain yield, yield components, leaf physiological traits, grain filling characteristics, and K accumulation dynamics. We hypothesized that split application of K in winter wheat enhances K and N uptake, delays leaf senescence, and improves photosynthetic performance, thereby boosting productivity. These effects are expected to vary between silty and sandy loam soils due to differences in nutrient dynamics and soil properties. Our objectives were to: (i) evaluate the impact of split K application on winter wheat grain yield; (ii) investigate its effects on the grain-filling process and flag leaf physiological characteristics; and (iii) examine K and N accumulation dynamics in aboveground plant organs under split K application in silty and sandy loam soils. The aim was to provide theoretical bases and technical support for high-yield and efficient wheat production.

## 2 Materials and methods

### 2.1 Experimental site

**Figure 1:**
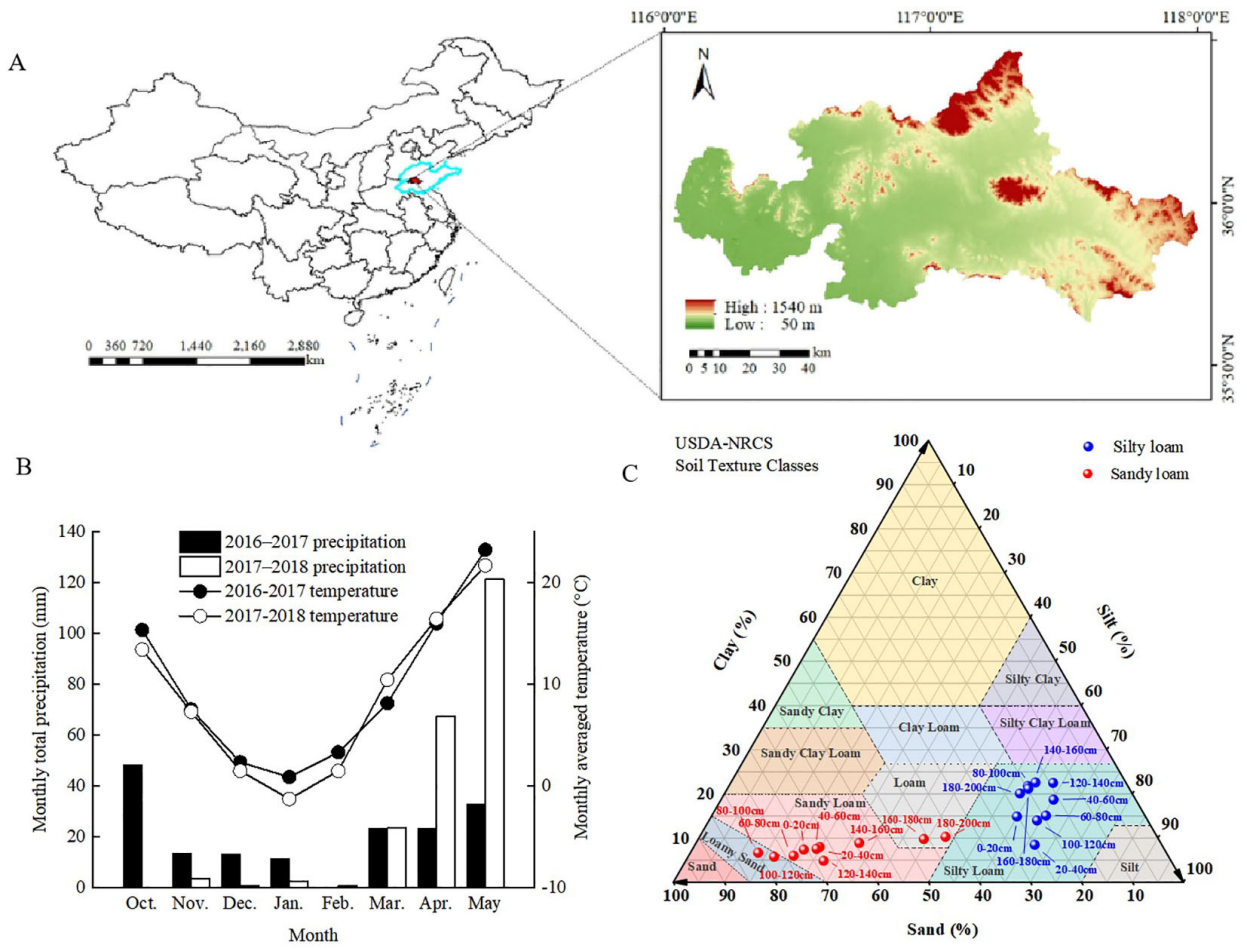
The location of the test area **(A)**, monthly total precipitation and monthly mean temperature for the growing seasons of winter wheat in 2016–2017 and 2017-2018 **(B)**, along with showing the percentages of texture for each particle size (Silt, Sand, and Clay) within the 0–200 cm depth of soil **(C)**.

We conducted field experiments in Daolang Town, Tai’an City (**Figure 1A**), Shandong Province (116°54’N, 36°12’E), during the 2016–2017 and 2017–2018 winter wheat-growing seasons. The two experimental fields, located 2 km apart, were characterized by silty loam and sandy loam soils, respectively. The region has a temperate continental monsoon climate, with an annual average temperature of 13.0–13.6°C and rainfall of 621.2–688.0 mm. The weather conditions during the experiment are shown in **Figure 1B**, and the soil properties before sowing are depicted in **Figure 1C** (soil depth ranging from 0 to 200 cm). According to the International Soil Texture Classification System, the primary soil types were classified as silt loam (0–200 cm soil layer texture: 20.07% sand, 60.01% silt, 17.93% clay) and sandy loam (0–200 cm soil layer texture: 64.95% sand, 27.17% silt, 7.8% clay), with parent material derived from loess. While before sowing, composite soil samples (0–20 cm depth) were collected from three five random points per plot to verify baseline soil fertility (**Table 1**). Wheat was sown in October and harvested in June of the following year, followed by immediate maize planting for October harvest.

**Table 1:** Soil nutrient status in the upper 20 cm soil layer before sowing in 2016–2017 and 2017–2018.

Year	Experimental plots	Organic matter	Total N	Hydrolysable N	Available P	Available K
		(%)	(g kg^-1^)	(mg kg^-1^)	(mg kg^-1^)	(mg kg^-1^)
2016–2017	Silty loam	1.16	1.18	111.06	45.98	168.06
	Sandy loam	1.00	0.91	101.45	37.14	91.05
2017–2018	Silty loam	1.15	1.18	112.08	45.49	142.62
	Sandy loam	0.88	1.04	107.20	36.99	89.64

### 2.2 Experimental design and planting material

The experiment employed a randomized complete block design with three replications. To ensure soil homogeneity, the experimental plots (2.4 m×20 m) were selected based on uniform topography, consistent prior crop management (maize residue incorporation), and pre-experiment soil analysis. The experiment was conducted in two soil types: silty loam and sandy loam. Three K application levels were established in both soils, which includes: no potassium (K0), 96 kg ha^-1^ (K1), and 120 kg ha^-1^ (K2). Two fertilization methods were implemented: T1 (100% basal application before sowing), and T2 (50% basal application before sowing and 50% topdressing at the jointing). Potassium chloride (KCl), containing 60% K_2_O, was used as the K fertilizer (**Table 2**). The winter wheat cultivar Shannong (SN29), widely cultivated in the Huang-Huai-Hai region, was selected as the experimental material due to its strong tillering ability, multi-spike morphology, lodging resistance, high yield potential, and adaptability to high-fertility soil in Shandong Province.

**Table 2:** K fertilizer application levels and methods under different soil types.

Soil type	Treatment	Total K_2_O application (kg ha^-1^)	K application period and proportion	
			Sowing (%)	Jointing (%)
Silty loam	K0	0	―	―
	K1T1	96	100	0
	K1T2	96	50	50
	K2T1	120	100	0
	K2T2	120	50	50
Sandy loam	K0	0	―	―
	K1T1	96	100	0
	K1T2	96	50	50
	K2T1	120	100	0
	K2T2	120	50	50

K0, no K fertilizer application; K1T1, basal application of 96 kg ha^-1^ K fertilizer; K1T2, basal application of 48 kg ha^-1^ K fertilizer, followed by application of 48 kg ha^-1^ K fertilizer at the jointing; K2T1, basal application of 120 kg ha^-1^ K fertilizer; K2T2, basal application of 60 kg ha^-1^ K fertilizer, followed by application of 60 kg ha^-1^ K fertilizer at the jointing.

### 2.3 Crop management

Urea fertilizer was applied as N fertilizer (containing 46% N), with the amount of 240 kg ha^-1^, which was applied in two installments (50% basal application and 50% topdressing at the jointing stage). Calcium superphosphate (containing 46% P_2_O_5_) was applied as P fertilizer, with an amount of 120 kg ha^-1^, which was applied as a base fertilizer before sowing. Topdressing was implemented using a fertilizer-water integrated sprinkler irrigation system. In cropping season 2016–17, both plots were sown on October 3; the silty loam was harvested on June 4, and the sandy loam was harvested on June 13, whereas in cropping season of 2017–18, both plots were sown on October 12 and harvested on June 5. Corn was the previous crop in the experimental field; corn stalks were crushed and returned to the field after harvest. The field implemented a unified management strategy for water, fertilizer, weeds, pests, and disease control, adhering to the local general control measures.

### 2.4 Measurement indicators and methods

#### 2.4.1 Flag leaf chlorophyll content (SPAD) and net photosynthetic rate (Pn)

The flag leaf chlorophyll content (SPAD) value and Pn of the flag leaf were measured using a Minolta SPAD-502 (Konica Minolta Sensing, Osaka, Japan) and Li-6400 portable photosynthesis system (Li-COR, USA) between 9:00 and 11:00 am in the early, middle, and late-filling stages of winter wheat. Each treatment involved measuring 30 flag leaves, which were tagged at anthesis and exhibited consistent growth.

#### 2.4.2 Physiological indexes of leaf senescence

The leaf samples (0.5 g) were ground in a frozen mortar with extraction media that had been precooled. The homogenates were centrifuged for 20 min at 12,000 rpm at 4°C. The filtrate was obtained as an enzyme extract for the determination superoxide dismutase (SOD) and catalase (CAT) activities of the flag leaf were determined by following the method of Beauchamp and Fridovich (1971); Aebi (1984). The malondialdehyde (MDA) content was determined by following the method of Du and Bramlage (1992). The soluble protein content (SPC) was determined by following the method of Bradford (1976) with some modifications.

#### 2.4.3 Grain weight increase and grain-filling rate

To assess grain filling characteristics, Wheat spikes were marked at 5, 10, 15, 20, 25, 30, 35, and 40 d after flowering in the 2016–2017 growing season, and at 7, 14, 21, 28, and 35 d after flowering in the 2017–2018 growing season. Each treatment marked 30 spikes in each period. Wheat spikes were dried and threshed, and 1000-grain weight (TGW) was determined. This process was repeated three times.

Logistic equation (Equation 1) was used to simulate the grain weight gain dynamics of winter wheat (Zhang et al., 2024).


(1) $$\begin{array}{c} W=\frac{A}{1+Be^{-Kt}} \end{array}$$

In Equation (1), W represents TGW, A represents the amount of growth termination, t is the number of days after flowering (DAF), and B, and K are fit parameters.


(2) $$\begin{array}{c} G=ABKe^{-Kt}{\left(1+Be^{-Kt}\right)}^{-2} \end{array}$$

In Equation (2), G represents the grain-filling rate, derived from the first derivative of the Logistic equation (Equation 1). When the grain-filling process reaches 99% of A, the actual end period of filling(*T3*) (Equation 3), the active grain-filling period (*D*) (Equation 4), average grain-filling rate (*G_mean_*) (Equation 5), and maximum grain-filling rate (*G_max_*) are calculated (Equation 6) while *T_max_* represents the time reaching a maximum grain-filling rate (Equation 7).


(3) $$\begin{array}{c} T3=\frac{ln\text{B}+4.59412}{K} \end{array}$$

(4) $$\begin{array}{c} D=\frac{lnB+2.19722}{K} \end{array}$$

(5) $$\begin{array}{c} G_{mean}=\frac{\text{KA}}{ln\text{B}+4.59512} \end{array}$$

(6) $$\begin{array}{c} G_{max}=\frac{\text{KA}}{4} \end{array}$$

(7) $$\begin{array}{c} T_{max}=\frac{ln\text{B}}{\text{K}} \end{array}$$

#### 2.4.4 The accumulation of K and the utilization efficiency of K fertilizer

The total K content of each organ in aboveground portions of plants was determined by concentrated sulfuric acid digestion and flame photometry (Jones and Case, 1990). The K accumulation in the aboveground part at maturity is equal to the sum of K accumulations in various organs.


(8) $$\begin{array}{c} K\;partial\;factor\;productivity\;\left(KPFP,\;kg\;kg^{-1}\right)=\frac{\text{Y}}{{\text{F}}_{K}} \end{array}$$

In Equation (8), where Y is the grain yield (kg ha^-1^),F_K_ is the K application amount (kg ha^-1^).


(9) $$\begin{array}{c} K\;agronomic\;efficiency\;\left(KAE,\;kg\;kg^{-1}\right)=\frac{Y_{K}-Y_{0K}}{{\text{F}}_{K}}\times 100 \end{array}$$

Where KAE is the agronomic efficiency of K fertilizer (kg kg^-1^), in Equation (9) Y_K_ is the grain yield (kg ha^-1^) in the K application area, Y_0K_ is the grain yield (kg ha^-1^) in the non-K application area, and F_K_ is the K application amount (kg ha^-1^).

#### 2.4.5 Nitrogen accumulation

The Kjeldahl method was used to determine N in soil and plants (Sáez-Plaza et al., 2013). The N accumulation in the aboveground part at maturity is equal to the sum of N accumulations in various organs.

#### 2.4.6 Grain yield and yield components

At the maturity stage, 30 wheat ears were randomly collected from each experimental plot to investigate the number of grains per spike (GN). Each plot was harvested from an area of 6 m^2^, threshed, and naturally air-dried until the grain moisture content reached approximately 12.5%. Dried grains were then weighed to calculate yield per hectare. Additionally, 1000 grains were randomly selected and weighed to determine TGW. Each treatment was repeated three times.

### 2.5 Data analysis

Analysis of variance was performed using SPSS 26.0 (SPSS Inc., Chicago, IL, USA). The means in different groups were compared by analysis of variance (ANOVA) followed by least significant difference test (for normally distributed data) or Kruskal-Wallis test (for not normally distributed data). A value of *P<*0.05 was considered statistically significant. Bar and line graphs were visualized using Origin 2023 (OriginLab, Northampton, MA, USA). Field map was drawn using ArcGIS software (version 10.4, ESRI, Redlands, US).

## 3 Results

### 3.1 K accumulation of aboveground plants

**Figure 2:**
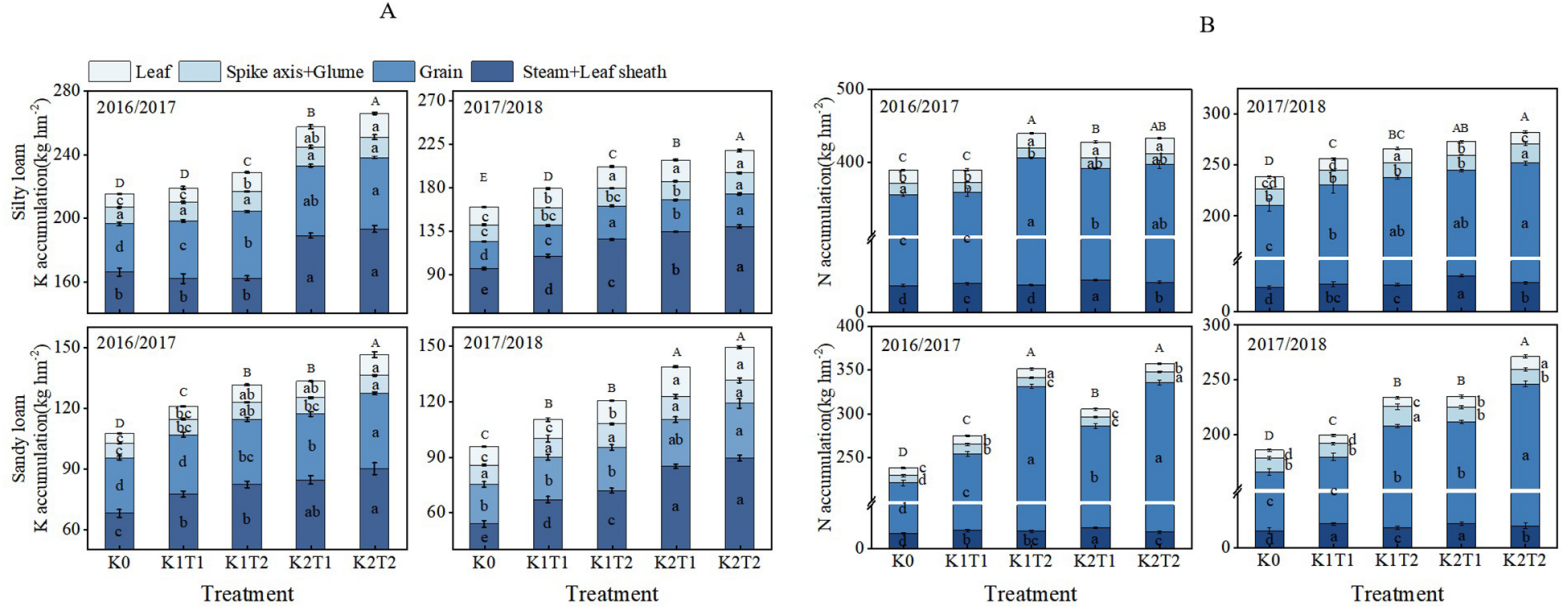
Differences in potassium **(A)** and nitrogen **(B)** accumulation in aboveground organs of plants under different treatments. K0, no K fertilizer application; K1T1, basal application of 96 kg ha^-1^ K fertilizer; K1T2, basal application of 48 kg ha^-1^ K fertilizer, followed by application of 48 kg ha^-1^ K fertilizer at the jointing; K2T1, basal application of 120 kg ha^-1^ K fertilizer; K2T2, basal application of 60 kg ha^-1^ K fertilizer, followed by application of 60 kg ha^-1^ K fertilizer at the jointing. The least significant difference (LSD) method was used for multiple comparisons. Lowercase and uppercase letters above the bars indicate significant differences (*P*<0.05) between treatments.

The growing season and K fertilizer level significantly influenced K accumulation in various organs of winter wheat, while the fertilization method notably affected K accumulation in the leaves and grains (**Table 3**). The split application of K enhanced K accumulation in the aboveground parts of plants compared to a single application. By analyzing different fertilization methods across both soil types, we found that in silty loam under K1, K accumulation in plants increased by an average of 8.61% compared to single K application (T1), while under K2, it increased by 4.00% (**Figure 2A**). In sandy loam, K accumulation in plants increased by 9.05% and 8.77% for K1 and K2 compared to the single K application (T1). In silty loam soils, the increase in K accumulation primarily occurred in the stem and leaf sheath, and grain while in sandy loam soils, the increased K accumulation was mainly observed in the grain (**Figure 2A**).

**Table 3:** Effects of year, fertilization method and K fertilizer level on K accumulation in aboveground parts of mature winter wheat.

Soil type	Source of variation	df	Steam+Leaf sheath	Leaf	Spike axis+Glume	Grain	K accumulation
Silty loam	Y	1	2746.31**	833.48**	322.02**	326.57**	1253.50**
	M	1	17.55**	19.62**	3.62ns	22.84**	42.93**
	L	2	339.28**	71.62**	12.51**	257.70**	508.82**
	Y × M	1	7.73*	0.29ns	0.01ns	1.60ns	3.43ns
	Y × L	2	48.08**	2.90ns	2.23ns	44.66**	24.81**
	M × L	2	5.55*	6.19*	1.74ns	11.84**	13.29**
	Y × M × L	2	6.57**	0.26ns	0.59ns	1.99ns	2.54ns
Sandy loam	Y	1	77.51**	253.10**	56.79**	80.20**	19.46**
	M	1	12.04**	17.62**	1.96ns	6.77*	27.22**
	L	2	266.10**	89.09**	4.40*	24.13**	268.13**
	Y × M	1	0.03ns	0.00ns	0.10ns	0.33ns	0.12ns
	Y × L	2	20.15**	11.47**	0.27ns	0.50ns	13.14**
	M × L	2	3.01ns	4.49*	1.00ns	2.74ns	6.89**
	Y × M × L	2	0.04ns	0.03ns	0.81ns	0.23ns	0.09ns

Y, year; M, fertilization methods; L, fertilizer level. Numbers in analysis of variance table represent F values; df, degrees of freedom. *Significance at *P*<0.05, **P<0.01; ns, non-significant, *P*>0.05.

Through analyzing K application regimes across silty loam and sandy loam soils, distinct patterns emerged. Compared to the zero-K control (K0), single applications increased two-year average K accumulation by 6.95% (K1) and 25.15% (K2) in silty loam, whereas split applications enhanced these values to 16.37% and 30.19%, respectively. Sandy loam exhibited stronger responses: single applications yielded 13.88% (K1) and 34.62% (K2) increases, which further rose to 24.19% and 46.31% with split applications. These results demonstrate that: (1) both application methods significantly promote K accumulation (*P*<0.05), (2) K2 consistently outperforms K1 across all treatments, and (3) sandy loam shows 1.3–1.8 times higher responsiveness than silty loam (**Figure 2A**).

### 3.2 N accumulation of aboveground plants

The N accumulation in various plant organs is significantly influenced by different growth seasons, K fertilizer levels, and fertilization methods (**Table 4**). N accumulation and its partitioning in different parts of the winter wheat varied with the application of various K fertilization methods (**Figure 2B**). At maturity stage, in silty loam soil under K1 conditions, the average N accumulation is significantly higher (*P*<0.05) compared to K2 when K was applied in splits rather than as a single application. While in sandy loam soil, compared to a single K application, split applications increased N accumulation by 22.66% and 16.24% for K1 and K2, respectively (**Figure 2B**). These findings suggest that the application of K in splits results in higher N accumulation compared to a single application, primarily due to increased N accumulation in the grains.

**Table 4:** Effects of year, fertilization method and K fertilizer level on N accumulation in aboveground parts of mature winter wheat.

Soil type	Source of variation	df	Steam+Leaf sheath	Leaf	Spike axis+Glume	Grain	N accumulation
Silty loam	Y	1	1759.61**	1097.76**	11.12**	3477.26**	4146.36**
	M	1	54.32**	7.29*	4.97*	32.96**	27.34**
	L	2	212.38**	40.55**	11.51**	66.11**	98.02**
	Y × M	1	1.34ns	0.60ns	2.87ns	6.86*	6.11*
	Y × L	2	3.9113ns	16.58**	2.09ns	0.30ns	0.11ns
	M × L	2	27.69**	22.98**	2.86ns	12.88**	14.39**
	Y × M × L	2	8.09**	2.25ns	3.36ns	10.56**	8.56**
Sandy loam	Y	1	20.92**	58.93**	449.97**	2332.17**	1879.94**
	M	1	64.00**	49.58**	29.71**	440.15**	362.81**
	L	2	138.82**	159.48**	56.39**	681.52**	722.98**
	Y × M	1	0.31ns	24.38**	17.45**	45.43**	29.72**
	Y × L	2	2.90ns	174.38**	16.77**	66.23**	52.21**
	M × L	2	18.24**	14.17**	13.15**	112.83**	94.34**
	Y × M × L	2	10.97**	7.32**	44.48**	17.56**	12.22**

Y, year; M, fertilization methods; L, fertilizer level. Numbers in analysis of variance table represent F values; df, degrees of freedom. *Significance at *P*<0.05, **P<0.01; ns, non-significant, *P*>0.05.

Furthermore, different K application rates revealed that in silty loam soil, a single K application increased the average N accumulation in plants by 3.83% and 12.28% for K1 and K2, respectively, compared to K0. In silty loam soil with split K applications, N accumulation increased by 12.34% and 14.92% for K1 and K2, respectively, compared to K0. In sandy loam soil, a single K application resulted in increases of 11.20% and 27.22% for K1 and K2, respectively, compared to K0. In sandy loam soil with split K applications, the two-year average N accumulation in plants increased by 36.60% and 47.88% for K1 and K2, respectively, compared to K0. These findings suggest that, regardless of whether K is applied as a single or split application, K2 is more effective than K1 in enhancing N accumulation (**Figure 2B**).

### 3.3 Photosynthetic characteristics of the flag leaf

**Figure 3:**
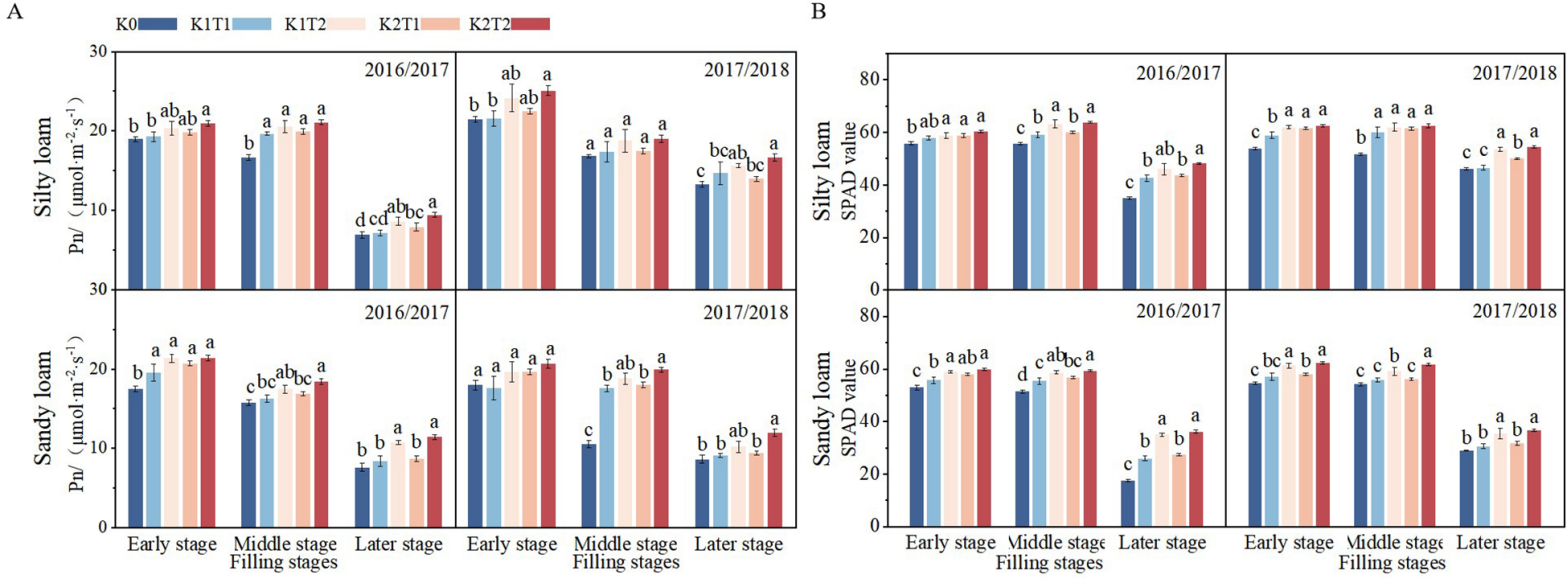
Differences in Net Photosynthetic rate **(A)** and SPAD values **(B)** of flag leaves among different treatments during the early, middle, and late stages of grain filling stage under different soil types. K0, no K fertilizer application; K1T1, basal application of 96 kg ha^-1^ K fertilizer; K1T2, basal application of 48 kg ha^-1^ K fertilizer, followed by application of 48 kg ha^-1^ K fertilizer at the jointing; K2T1, basal application of 120 kg ha^-1^ K fertilizer; K2T2, basal application of 60 kg ha^-1^ K fertilizer, followed by application of 60 kg ha^-1^ K fertilizer at the jointing. The least significant difference (LSD) method was used for multiple comparisons. Lowercase and uppercase letters above the bars indicate significant differences (*P*<0.05) between treatments.

The growth season, fertilization method, and K fertilizer level all have a significant (*P*<0.05) impact on both the SPAD value and Pn during the grain filling stage (**Table 5**). Winter wheat flag leaf SPAD and Pn variation during grain filling are similar across both soil types. The SPAD value remains high during the early and middle grain filling stages, but decline at later stage (**Figure 3A**). In silty loam soil, the split application of K1 increased the SPAD value and Pn of flag leaf during the filling stage by 6.66% and 10.37%, respectively, compared to a single K application. Similarly, the split application of K2 increased the SPAD value and Pn by 5.19% and 11.78%, respectively, relative to a single application. In sandy loam soil, the split application of K1 increased the SPAD value and Pn of the flag leaf during the filling stage by 12.64% and 12.50%, respectively, compared to a single K application. Similarly, the split application of K2 increased the SPAD value and Pn by 12.23% and 14.62%, respectively, relative to a single application (**Figures 3A, B**).

**Table 5:** Effects of year, fertilization method, and K fertilizer level on SPAD and Pn values during the early, middle, and late stages of grain filling.

Soil type	Source of variation	df	SPAD value			Pn		
			Early stage	Middle stage	Later stage	Early stage	Middle stage	Later stage
Silty loam	Y	1	3.36*	6.40*	220.78**	102.44**	33.69**	1420.77**
	M	1	5.30*	10.97**	38.82**	17.87**	12.17**	45.71**
	L	2	63.92**	91.09**	97.09**	13.91**	46.98**	39.58**
	Y × M	1	0.31ns	2.38ns	1.15ns	2.85ns	0.28ns	1.16ns
	Y × L	2	9.63**	5.80**	10.19**	0.78ns	9.86**	0.64ns
	M × L	2	1.58ns	2.86ns	9.87**	4.47*	3.09*	12.44**
	Y × M × L	2	0.98ns	0.62ns	1.40ns	0.71ns	0.07ns	1.14ns
Sandy loam	Y	1	14.48**	9.16**	126.12**	5.39**	21.61**	7.40**
	M	1	30.12**	29.85**	90.20**	8.14**	27.72**	49.29**
	L	2	71.87**	62.34**	158.86**	27.48**	293.88**	41.14**
	Y × M	1	1.97ns	1.36ns	7.06ns	0.08ns	0.13ns	1.22ns
	Y × L	2	0.17ns	2.78ns	37.00**	4.31*	137.63**	1.85ns
	M × L	2	7.63**	7.72**	22.55**	2.89ns	7.51**	14.10**
	Y × M × L	2	0.69ns	1.20ns	1.79ns	0.02ns	0.13ns	0.94ns

Pn stands for net photosynthetic rate. Y, year; M, fertilization methods; L, fertilizer level. Numbers in analysis of variance table represent F values; df, degrees of freedom. *Significance at *P*<0.05, **P<0.01; ns, non-significant, *P*>0.05.

### 3.4 Stress resistance characteristics

In silty loam, the K method significantly influenced catalase (CAT) content during the 0–10 days after flowering (DAF), malondialdehyde (MDA) content during the 20–30 DAF period, and soluble protein content throughout the 0–30 DAF interval. In sandy loam, the fertilization method significantly affected superoxide dismutase (SOD), MDA, and soluble protein content during the 20–30 DAF period. Additionally, levels of K fertilizer significantly influenced leaf senescence characteristics. Furthermore, a significant interaction was observed between the fertilization method and K fertilizer level regarding MDA and soluble protein content during the 20–30 DAF period (**Table 6**).

**Table 6:** Effects of fertilization method, K fertilizer level, and their interactions on flag leaf senescence characteristics under different soil conditions.

Soil type	Source of variation	df	SOD				CAT				MDA				Soluble protein			
			0d	10d	20d	30d	0d	10d	20d	30d	0d	10d	20d	30d	0d	10d	20d	30d
Silty loam	M	1	3.04 ns	0.84 ns	0.09 ns	1.79 ns	8.85 *	8.19 *	3.82 ns	5.61 ns	14.78 *	2.26 ns	232.16 **	101.08 **	9.34 *	107.80 **	34.49 **	54.27 **
	L	2	4.63 ns	1.89 ns	7.55 *	12.94 *	10.39 *	14.85 **	5.59 *	12.29 *	24.35 **	5.15 *	104.10 **	81.07 **	32.19 **	262.07 **	55.67 **	48.79 **
	M × L	2	1.19 ns	0.21 ns	0.02 ns	0.45 ns	3.23 ns	3.58 ns	1.54 ns	1.92 ns	8.75 *	0.99 ns	59.98 **	26.95 **	2.92 ns	27.56 **	22.34 **	13.92 **
Sandy loam	M	1	2.95 ns	1.29 ns	10.02 *	20.14 **	14.93 *	5.42 ns	8.14 *	3.81 ns	2.77 ns	3.80 ns	34.44 **	21.87 **	1.03 ns	13.81 ns	38.43 **	89.95 **
	L	2	10.18 *	8.81 *	50.68 **	44.60 **	54.51 **	15.87 **	54.31 **	20.88 **	8.88 *	4.50 *	78.12 **	524.12 **	63.19 **	98.92 **	238.30 **	81.91 **
	M × L	2	0.85 ns	0.53 ns	3.28 ns	8.81 *	6.72 *	1.67 ns	5.34 ns	1.66 ns	0.82 ns	1.11 ns	9.11 *	16.71 **	6.94 ns	4.06 ns	12.26 *	25.77 **

SOD, superoxide dismutase; CAT, catalase; MDA, malondialdehyde. M, fertilization methods; L, fertilizer level. The number in the analysis of variance table represents the F value; df represents degrees of freedom; *Significant at *P*<0.05; **Significant at *P*<0.01; ns, non- significant, *P*>0.05.

**Figure 4:**
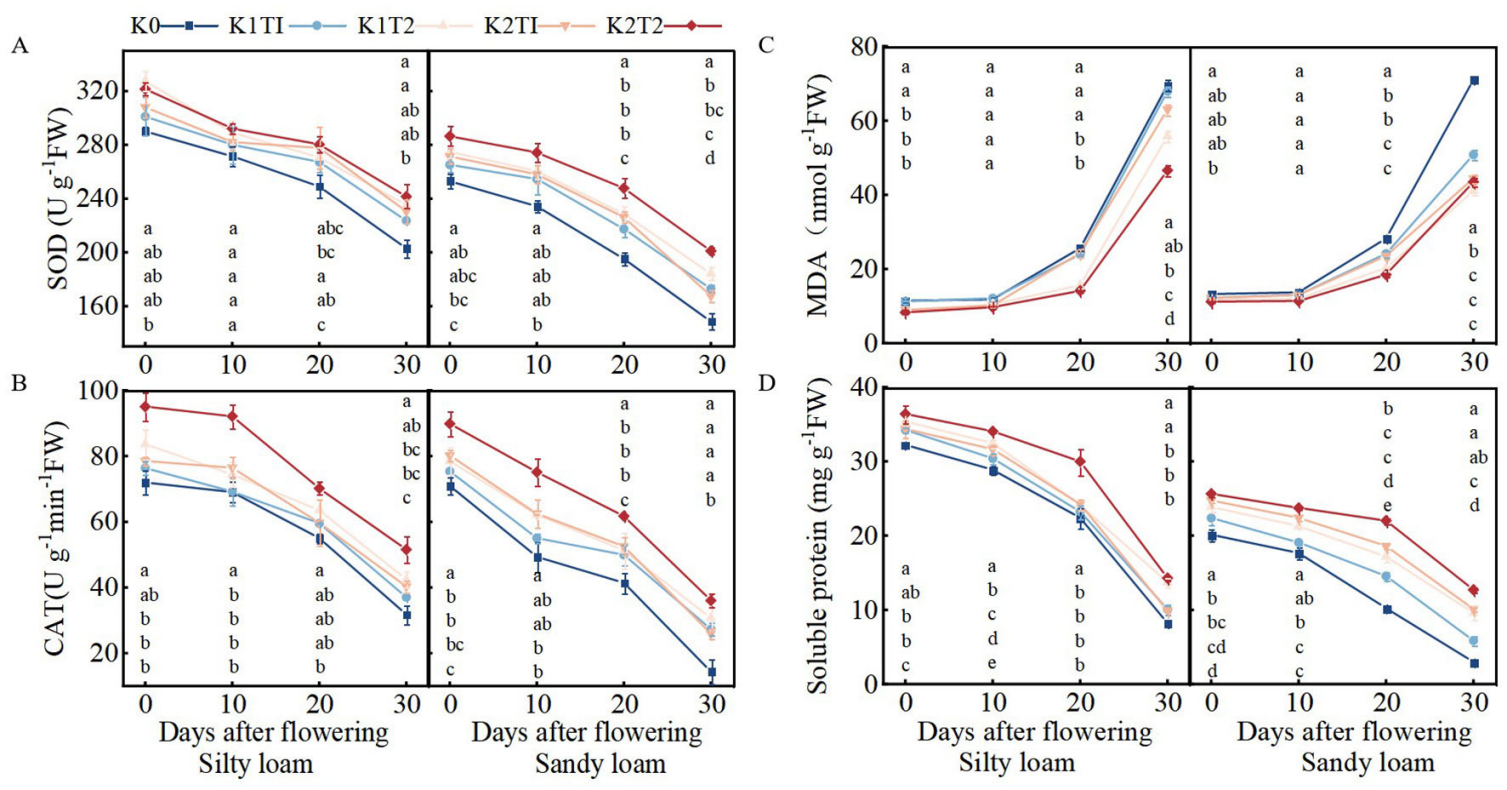
Changes in SOD **(A)** and CAT activities **(B)**, MDA content **(C)**, and soluble protein content **(D)** in flag leaves of winter wheat from 0 to 30 days after flowering. K0, no K fertilizer application; K1T1, basal application of 96 kg ha^−1^ K fertilizer; K1T2, basal application of 48 kg ha^−1^ K fertilizer, followed by application of 48 kg ha^−1^ K fertilizer at the jointing; K2T1, basal application of 120 kg ha^−1^ K fertilizer; K2T2, basal application of 60 kg ha^−1^ K fertilizer, followed by application of 60 kg ha^−1^ K fertilizer at the jointing. The least significant difference (LSD) method was used for multiple comparisons. Lowercase and uppercase letters above the bars indicate significant differences (*P*<0.05) between treatments.

In silty loam soil, when the K application rate is K1, split K application significantly increases the soluble protein content at 10 to 30 DAF compared to single K application, while also significantly reducing MDA content between 20 and 30 DAF. At the K2 application rate, split K application significantly increases CAT activity at 0–10 and 30 DAF, as well as the soluble protein content from 0 to 30 DAF, and significantly decreases MDA content between 20 and 30 DAF compared to single K application. In sandy loam soil, at the K1 application rate, split K application significantly increases the soluble protein content between 20 and 30 DAF and significantly decreases MDA content during the same period compared to single K application. At the K2 application rate, split K application significantly improves CAT activity at 20 DAF, SOD activity, and the soluble protein content between 20 and 30 DAF, while also significantly reducing MDA content at 20 DAF compared to single K application (**Figures 4A–D**).

In general, compared to single K application, split K application delayed the flag leaves senescence in winter wheat on silty loam soil from 0–30 DAF, and also delayed the senescence of flag leaves in winter wheat on sandy loam soil from 20–30 DAF. When the K application method was the same, K2 showed the best effect.

### 3.5 Grain-filling characteristics

For the same soil texture, compared to single application, the split K application prolonged the active grain-filling period (*D*) and the actual end period of filling (*T3*), but it also reduced the mean grain-filling rate (*G_mean_*) and the maximum grain-filling rate (*G_max_*). In different soil textures, compared to K0, the application of K prolonged D and T3 (**Table 7**).

**Table 7:** The fitting equation and parameters of soil texture and grain dry matter accumulation under different fertilization treatments.

Year	Experimental field	Treatment	Model of grain-filling process	*R^2^*	*G_mean_* (mg grain^–1^d^–1^)	*G_max_* (mg grain^–1^d^–1^)	*T_max_ (d)*	*D* (d)	*T3* (d)
2016–2017	Silty loam	K0	W=46.05/(1 + 47.53e^-0.22t^)	0.998	1.21	2.56	17.35	27.22	37.99
		K1T1	W=45.63/(1 + 47.91e^-0.22t^)	0.998	1.18	2.49	17.72	27.77	38.75
		K1T2	W=45.22/(1 + 34.99e^-0.21t^)	0.998	1.14	2.32	17.33	28.04	39.73
		K2T1	W=46.28/(1 + 52.05e^-0.23t^)	0.998	1.25	2.67	17.10	26.60	36.97
		K2T2	W=46.06/(1 + 37.76e^-0.21t^)	0.998	1.17	2.41	17.34	27.84	39.29
	Sandy loam	K0	W=46.83/(1 + 38.53e^-0.21t^)	0.984	1.21	2.49	17.18	27.51	38.79
		K1T1	W=47.69/(1 + 38.79e^-0.21t^)	0.996	1.21	2.51	17.40	27.86	39.26
		K1T2	W=49.52/(1 + 37.72e^-0.21t^)	0.997	1.26	2.59	17.34	27.88	39.28
		K2T1	W=49.29/(1 + 33.14e^-0.20t^)	0.995	1.21	2.46	17.57	28.59	40.62
		K2T2	W=49.84/(1 + 29.56e^-0.19t^)	0.998	1.20	2.40	17.61	29.04	41.51
2017–2018	Silty loam	K0	W=47.68/(1 + 32.58e^-0.19t^)	0.999	1.12	2.26	18.36	29.95	42.59
		K1T1	W=47.93/(1 + 25.91e^-0.17t^)	0.997	1.06	2.07	18.82	31.53	45.39
		K1T2	W=48.00/(1 + 26.33e^-0.17t^)	0.990	1.06	2.08	18.85	31.55	45.32
		K2T1	W=48.32/(1 + 31.09e^-0.19t^)	0.999	1.15	2.31	17.95	29.42	41.94
		K2T2	W=49.31/(1 + 28.85e^-0.18t^)	0.998	1.12	2.23	18.59	30.74	43.99
	Sandy loam	K0	W=52.17/(1 + 40.99e^-0.19t^)	0.996	1.22	2.53	19.16	30.50	42.87
		K1T1	W=51.00/(1 + 40.82e^-0.19t^)	0.999	1.19	2.47	19.17	30.53	42.92
		K1T2	W=51.20/(1 + 35.46e^-0.19t^)	0.995	1.18	2.41	18.96	30.63	43.37
		K2T1	W=52.94/(1 + 29.06e^-0.18t^)	0.995	1.23	2.44	18.27	30.19	43.19
		K2T2	W=53.87/(1 + 25.74e^-0.18t^)	0.992	1.20	2.36	18.52	31.04	44.71

In the grain filling equation, W and t represent 1000-grain weight and days after flowering, respectively. *R^2^* represents the coefficient of determination of the equation. *G_mean_* represents the average grain-filling rate, *G_max_* represents the maximum grain-filling rate, *T_max_* represents the time reaching a maximum grain-filling rate, *D* represents the active grain-filling period, and *T3* represents the actual end period of filling. K1T1, basal application of 96 kg ha^−1^ K fertilizer; K1T2, basal application of 48 kg ha^−1^ K fertilizer, followed by application of 48 kg ha^−1^ K fertilizer at the jointing; K2T1, basal application of 120 kg ha^−1^ K fertilizer; K2T2, basal application of 60 kg ha^−1^ K fertilizer, followed by application of 60 kg ha^−1^ K fertilizer at the jointing.

### 3.6 Yield components and K fertilizer utilization

**Figure 5:**
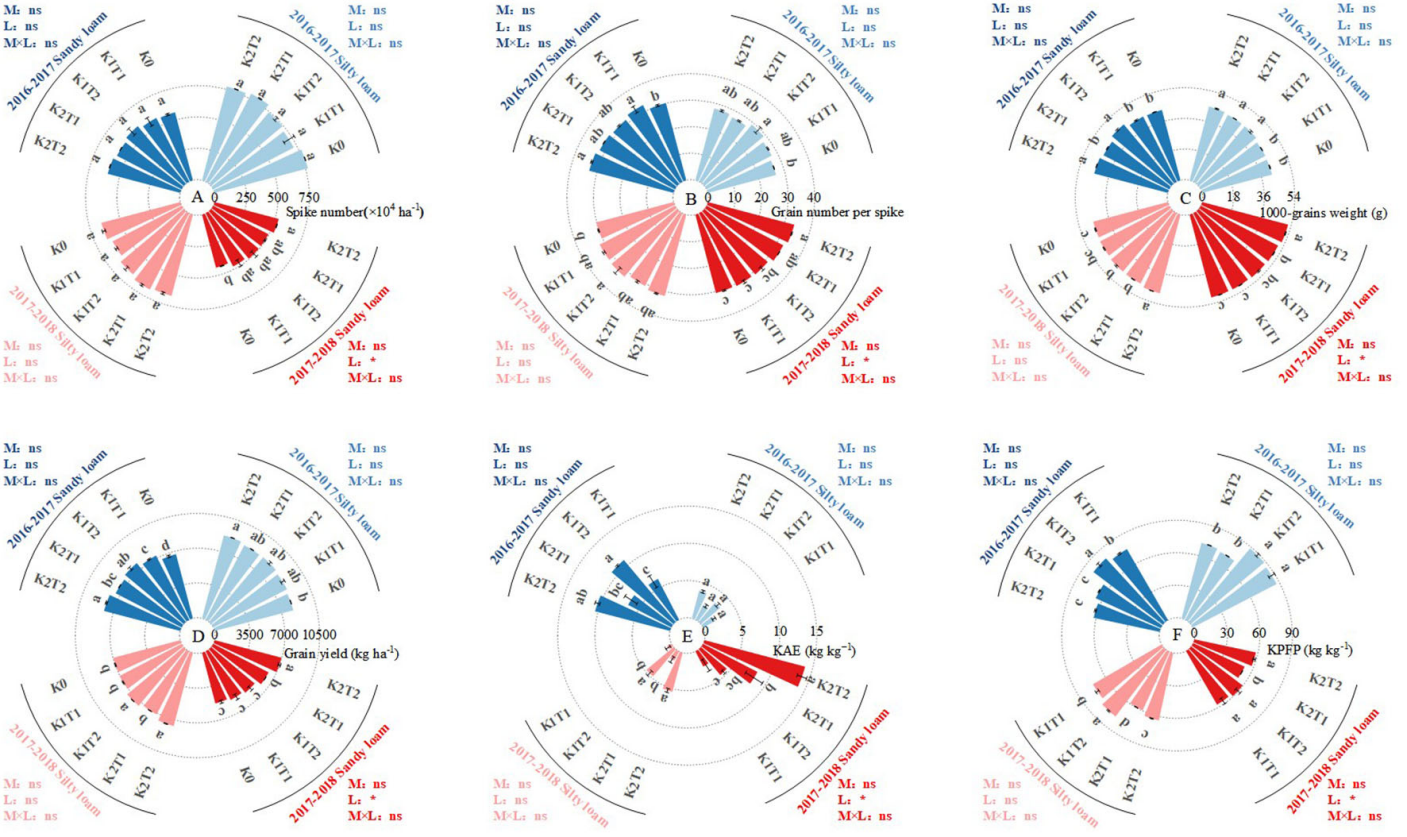
Yield components and difference in K fertilizer utilization efficiency. Spike number **(A)**; Grain number per spike **(B)**; 1000-grain weight **(C)**; grain yield **(D)** KAE, K fertilizer agronomic efficiency **(E)**; KPFP, partial factor productivity of K fertilizer **(F)**. K0, no K fertilizer application; K1T1, basal application of 96 kg ha^-1^ K fertilizer; K1T2, basal application of 48 kg ha^-1^ K fertilizer, followed by application of 48 kg ha^-1^ K fertilizer at the jointing; K2T1, basal application of 120 kg ha^-1^ K fertilizer; K2T2, basal application of 60 kg ha^-1^ K fertilizer, followed by application of 60 kg ha^-1^ K fertilizer at the jointing. The least significant difference (LSD) method was used for multiple comparisons. Lowercase above the bars indicate significant differences (*P*<0.05) between treatments.

The split application of K exhibited a significant response in terms of grain yield, 1000-grain weight (TGW), and agronomy efficiency of K fertilizer (KPFP) in both soil types for both growing seasons (**Figures 5C–E**). The K fertilizer level had no significant effect on the number of spikes per hectare and grain per spike only in silty loam soil (**Figures 5A, B**). For silty loam soil, compared to a single K application, the split application of K1 led to an average increase of 2.35% in TGW, 3.70% in grain yield, 2.90 kg kg^-1^ in KAE, and 2.83 kg kg^-1^ in KPFP. In the K2 treatment, the respective increases were 2.12% in TGW, 4.48% in grain yield, 2.80 kg kg^-1^ in KAE, and 2.79 kg kg^-1^ in KPFP. In contrast, in sandy loam soil, under the K1 treatment, the split application of K resulted in an average increase of 3.31% in TGW, 5.02% in grain yield, 3.40 kg kg^-1^ in KAE, and 3.45 kg kg^-1^ in KPFP compared to a single application. Under the K2 treatment, the respective increases were 2.41% in TGW, 8.60% in grain yield, 4.85 kg kg^-1^ in KAE, and 0.95 kg kg^-1^ in KPFP (**Figures 5C–F**).

In a two-year experiment analyzing various K application rates across both soil types, the results indicated that the sole application of K1 did not significantly increase grain yield compared to K0 in either soil type. While the sole application of K2 resulted in a 3.32% and 2.08% increase in TGW, and a 2.91% and 15.21% increase in grain yield relative to K0 for silty loam soil and sandy loam soil, respectively. In both soil types, split applications of K improved TGW and grain yield compared to K0. In silty loam, K1 increased TGW and yield by 3.12% and 5.26%, respectively, while K2 increased them by 5.50% and 7.51%. In sandy loam, K1 enhanced TGW and yield by 3.84% and 11.89%, respectively, with K2 showing greater increases of 4.16% and 25.21% (**Figures 5C, D**).

### 3.7 Correlation analysis

**Figure 6:**
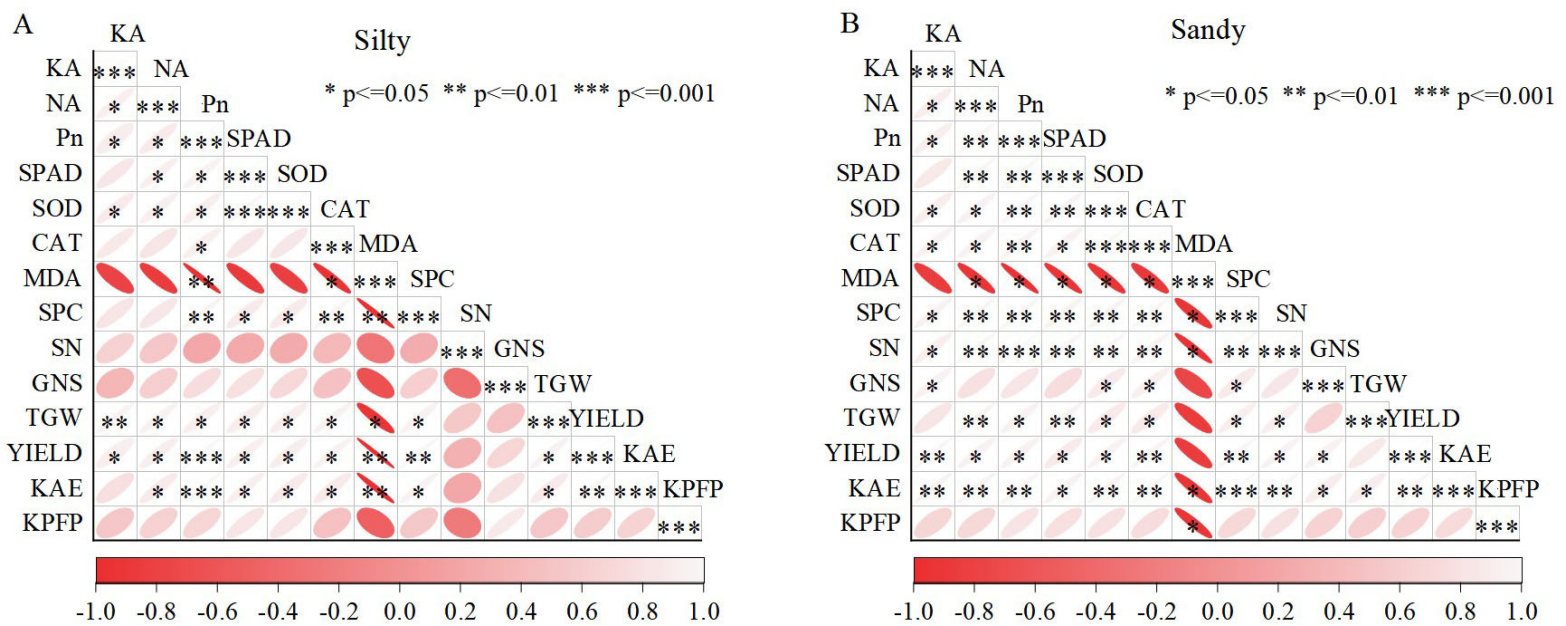
Correlation between yield, K fertilizer use efficiency, and physiological parameters under different soil conditions for silty **(A)** and sandy **(B)** soils. KA, K accumulation of aboveground plants; NA, N accumulation of aboveground plants; Pn, net photosynthetic rate; SPAD, chlorophyll SPAD value; SOD, superoxide dismutase; CAT, catalase; SPC, soluble protein; MDA, malondialdehyde; SN, number of spikes; GNS, per spike; TGW, 1000-grain weight; YIELD, grain yield; KAE, K fertilizer agronomic efficiency; KPFP, partial factor productivity of K fertilizer.

**Figure 7:**
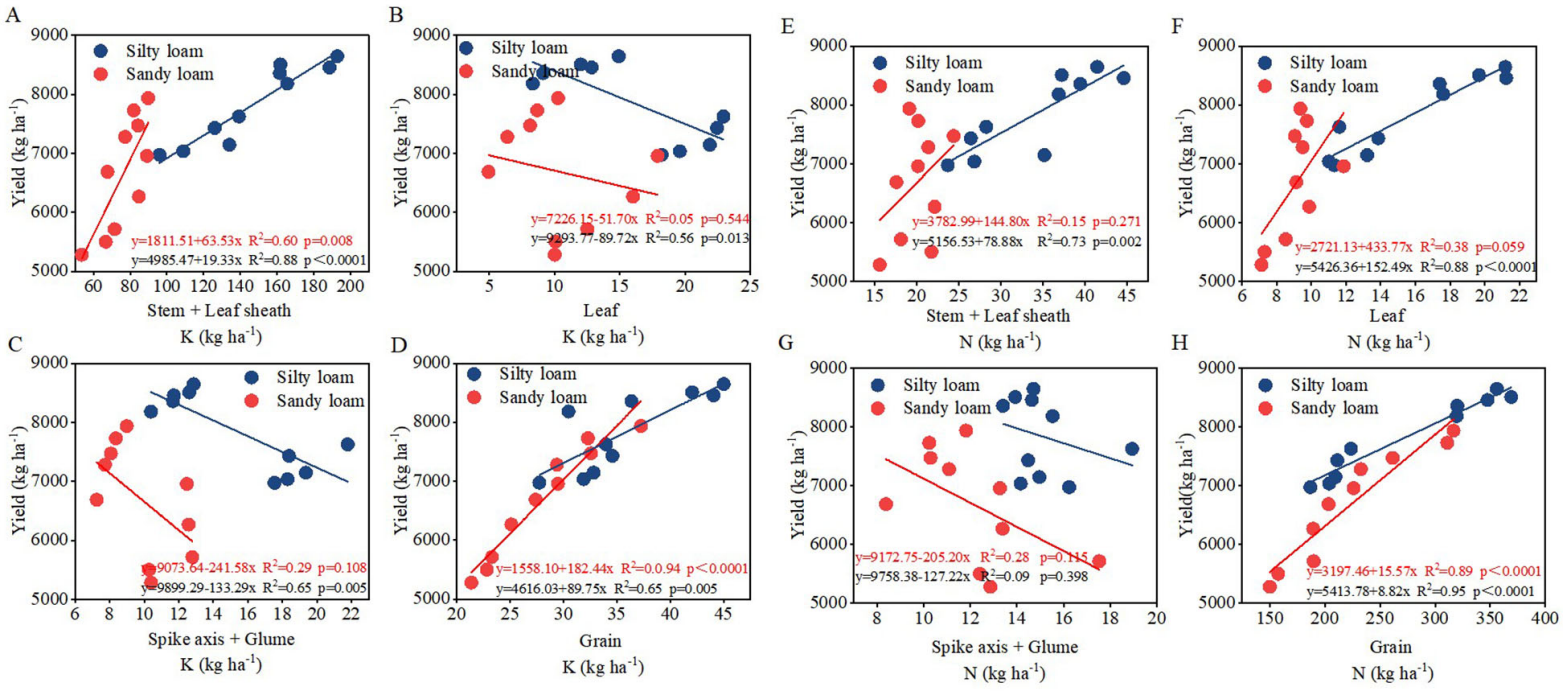
Relationship between K content in various organs and grain yield **(A–D)** and N content in various organs and grain yield **(E–H)** for silty loam (blue labels) and sandy loam (red labels). y indicates the regression equation, R^2^ represents the correlation coefficient, and p denotes the statistical significance of the regression analysis.

Correlation analysis revealed that the net photosynthetic rate (Pn) had a significant impact on N accumulation, K accumulation, leaf senescence characteristics, 1000-grain weight, and KAE. A significant positive correlation was observed between plant N accumulation and K accumulation, with both parameters significantly influencing yield (**Figure 6**). Notably, there is an extremely significant linear relationship between the accumulation of N and K in grains and yield (*P*<0.01) (**Figures 7A–H**); simultaneously, there is also an extremely significant linear relationship between K accumulation in stems and leaf sheaths and yield (*P*<0.01) (**Figures 7A–H**).

## 4 Discussion

### 4.1 Response of K absorption and distribution to split K application

Our study revealed that split K application significantly increased K accumulation in stems, leaf sheaths, leaves, and grains of winter wheat grown in silt loam soil, compared to a single K application, while the increase in sandy loam soil the increase was primarily observed in leaves and grains. These results are consistent with recent findings by Zang et al. (2024). Notably, wheat plants in sandy loam exhibited higher K uptake capacity than those in silt loam across all K application rates, with split K application proving superior to single application in both soil types. These observed differences likely reflect distinct K dynamics between the two soil types. Silt loam, characterized by its higher clay content and illite/vermiculite dominated mineralogy (Krauss et al., 2017; Bern and Yesavage, 2018), exhibits stronger K fixation into non-exchangeable forms, thereby reducing plant-available K. In contrast, sandy loam, dominated by montmorillonite and lower clay content, results in weaker K fixation capacity, maintaining higher levels of water-soluble and exchangeable K (Shaviv et al., 1985). The split K application strategy appears to effectively address K fixation in silt loam while simultaneously compensating for late-season K deficiency in sandy loam. Additionally, the greater sand fraction in sandy loam improves soil aeration and promotes root development, thereby facilitating K uptake (Corrêa et al., 2018).

### 4.2 Response of photosynthetic characteristics to split K application

The significant improvements in SPAD values and net photosynthetic rate (Pn) under split K application, particularly during the grain-filling phase (**Figures 3A, B**), demonstrated a strong positive correlation with enhanced K uptake (**Figure 6**). Potassium plays fundamental roles in multiple physiological processes, including cell elongation, enzyme activation, osmotic regulation, stomatal movement, and photosynthesis efficiency (Zhang et al., 2012). Our findings confirm that optimized K application through split applications enhances both chlorophyll content and photosynthetic performance (Verspreet et al., 2013; Wang et al., 2020).

Notably, split K application improved flag leaf photosynthetic capacity in both silt loam and sandy loam soils (Hu et al., 2021; Zhang et al., 2023), with more significant effects in sandy loam (**Figure 3**). The delayed enhancement of Pn during late grain filling stages suggests that split applications provide sustained K availability when needed most, effectively preventing late-season K deficiency during critical reproductive growth phases. This temporal synchronization between K supply and crop demand likely explains the superior photosynthetic performance observed under split application regimes.

### 4.3 Response of stress resistance to split K application

Delaying leaf senescence is critical for yield improvement (Zentgraf et al., 2004; Lim et al., 2007; Thomas, 2013; Lu et al., 2017). Plants mitigate reactive oxygen species (ROS) induced toxicity through antioxidant defense systems, particularly via the coordinated action of superoxide dismutase (SOD) and catalase (CAT), which maintain cellular homeostasis (Li and Kim, 2021). Malondialdehyde (MDA), a byproduct of membrane lipid peroxidation, serves as a reliable biomarker for oxidative stress and senescence progression (Ayala et al., 2014; Bresson et al., 2018). Simultaneously, soluble protein content has been established as a key indicator of plant stress response, with its accumulation directly correlating with delayed senescence and improved stress tolerance (Gupta et al., 2020).

Notably, both K deficiency and excessive K application can induce leaf senescence (Santos, 2001; Li et al., 2012; Wang et al., 2012; Du et al., 2019), while optimized K application delays it. Our results (**Figure 4C**) reveal that split K application significantly enhanced SOD and CAT activities, increased soluble protein content, and reduced MDA accumulation. These physiological responses collectively demonstrate improved ROS scavenging capacity and reduced membrane lipid peroxidation damage (Das and Roychoudhury, 2014). The observed effects were particularly pronounced in sandy loam soils, suggesting that split K application represents an effective strategy for delaying winter wheat leaf senescence, especially in coarser-textured soils. One possible explanation is that sandy loam soil, dominated by montmorillonite minerals with low clay content, exhibits weak potassium-fixing capacity and maintains high levels of readily absorbable exchangeable K+ (Shaviv et al., 1985). The uptake of exchangeable K+ reduces reactive oxygen species (ROS) generation in plants, decreases nicotinamide adenine dinucleotide phosphate (NADPH) oxidase activity, and sustains photosynthetic electron transport activity to assist in lowering ROS levels (Foyer, 2018). This process effectively alleviates oxidative damage to membrane systems, providing evidence for efficient potassium utilization and enhanced crop stress tolerance in sandy loam soils.

### 4.4 Response of yield and KUE to split K application

Potassium utilization efficiency (KUE) primarily depends on optimal K uptake (Geng et al., 2020). Our study demonstrated that the yield improvement from effect of K fertilizer could be attributed to improved stress tolerance and synergistic N-K absorption, with split K application further amplified these benefits. This improvement may be explained by the ability of split applications to maintain stable and sustained rhizospheric K availability. This avoids temporary soil K saturation, reduces leaching losses, and optimizes root absorption capacity during critical growth phases (Johnson et al., 2022).

In northern China’s wheat regions, split K application maximizes yield when soil available K ranges between 150–180 mg kg^-1^ but provides pronounced yield benefits (<90 mg kg^-1^ available K) (Ma et al., 2022). This aligns with previous studies that K fertilizer response correlates inversely with soil K status; such as lower inherent K levels result in greater yield increases (Fang et al., 2023). Our results corroborate this pattern: showing split K application increased yield by 4.09% in silt loam (average available K: 155.34 mg kg^-1^) and 6.81% in sandy loam (available K: 90.35 mg kg^-1^), highlighting greater yield potential in K-deficient sandy soils. Split K application predominantly enhanced yield by increasing grain weight rather than spike number, a trend more evident in sandy loam. Furthermore, split applications significantly improved KAE and KPFP compared to single applications. These findings underscore the importance of adopting soil-specific K management strategies that account for differential K availability across soil types.

## 5 Conclusions

Split application of K fertilizer enhanced photosynthetic capacity (particularly during the late grain-filling stage) by promoting synergistic uptake of K and N. It alleviated oxidative stress to delay leaf senescence while extending both the effective grain-filling period and the actual end of the filling stage. These physiological improvements ultimately increased TGW, leading to concurrent improvements in yield, KAE, and KPFP. Under the same K application rate, split application conferred more pronounced benefits in sandy loam soil, significantly enhancing these parameters compared to silty loam soil in the Huang-Huai-Hai wheat-growing region. For optimal results in both sandy loam and silty loam soils of this region, we recommend applying 120 kg K_2_O ha^-1^ in two splits. This fertilization strategy provides a robust basis for K management in agricultural production, emphasizing the critical role of temporal nutrient distribution in balancing crop physiological demands and soil-specific constraints. This study elucidated the efficiency-enhancing mechanisms of split K fertilization in the Huang-Huai-Hai wheat region. However, further research is needed to optimize integrated management strategies i.e., water-fertilizer coupling and cultivar selection and to evaluate the long-term effects of split K application, including soil K pool dynamics and sustainable productivity. Future research should focus on deciphering the molecular underlying K’s effects while systematically assessing its agronomic benefits through long-term field experiments.

## Data Availability

The original contributions presented in the study are included in the article/supplementary material. Further inquiries can be directed to the corresponding author.
